# Logical Gaps in the Approximate Solutions of the Social Learning Game and an Exact Solution

**DOI:** 10.1371/journal.pone.0115706

**Published:** 2014-12-29

**Authors:** Wenjie Dai, Xin Wang, Zengru Di, Jinshan Wu

**Affiliations:** School of Systems Science, Beijing Normal University, Beijing, P.R. China; Hong Kong Baptist University, China

## Abstract

After the social learning models were proposed, finding solutions to the games becomes a well-defined mathematical question. However, almost all papers on the games and their applications are based on solutions built either upon an ad-hoc argument or a twisted Bayesian analysis of the games. Here, we present logical gaps in those solutions and offer an exact solution of our own. We also introduce a minor extension to the original game so that not only logical differences but also differences in action outcomes among those solutions become visible.

## Introduction

The original version of social learning game (see [1], [2] for an introduction and a short review) is a problem with 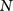 learners in which each learner (denoted as learner 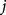) attempts to identify and act accordingly to the true status of a world (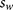), which is either in a state 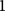 with probability 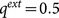 or in another state 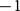 with probability 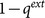, from observing her own private signals (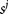) and all previous learners' actions (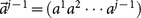) but without explicitly knowing the previous learners' private signals (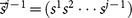). In the game it is assumed that the private signal received by each learner has a probability 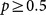 to be the true status of the world. It is usually required that one learner takes an action in every round. Usually, the turn order of learners' action is externally given. A learner receives a positive payoff (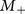) when her action is the same as the status of the world, and a negative payoff (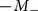, here 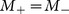) otherwise.

This model of observational social learning was proposed to describe herd behaviors such as formation of fads, fashion, or culture convention [3], [4]. For example, in deciding to purchase an iPhone or an Android phone, although personal opinions about the quality and features of the various phones are important, the choices of friends both locally and on social media, are at least, equally important, or sometimes even more important as personal opinions. Of course, in this case of cellphone purchasing, following friends might lead to a decision that one will later be not happy with. The question of whether following group wisdom [5] leads to better or worse decisions has been actively investigated.

It has been shown [3], [4] that in this typical setup there is an information cascade which can lead to either the proper status of the world or the wrong status even when all of the learners are fully rational. After the cascade happens, the rest of the learners choose the same action. This model and the implied cascade phenomenon even for fully rational learners are regarded to be to some degree a relevant model of dynamics of public opinion and formation of fashion and fads [3], [4]. To use this model to describe real-world phenomena, we need a mathematical theory to calculate action outcomes of every learner in this game.

One key problem in doing so for a learner at the 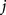
th place in the social learning game is how to determine the probability of world's status being 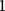 (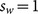), given the historical record of previous learner's action 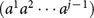 and her private signal 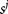, 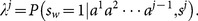
(1)Once a learner knows precisely this probability, she can always make an informed decision. Under the assumption that all other learners are as rational and capable as the 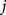
th learner herself, finding the right formula to calculate 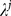 such that she will obtain the maximum payoff is a well-defined mathematical problem.

An exact solution of this mathematical problem refers to a fully rational solution of the above problem from an ideal learner with potentially infinite capability of mathematical calculation. However, except in the case where the private signal is also open to the public, there is no exact procedure to calculate this 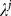 in the literature.

A common method of avoiding the calculation of 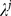 for the 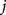
th learner is to count the number of actions with an observed value of 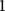 (denoted as 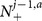) and the number of actions with an observed value of 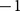 (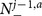) in the previous 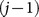 actions and then to act in concert with the majority after including her own private signals. We call this technique the blind action-counting approach and denote the calculated probability as 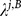 in the following. Quite often in theoretical analysis of the social learning game, one focuses on the phenomenon of “cascading”: After observing certain number of previous actions, the remaining learners choose the same action regardless of their private signals. Using the blind action-counting 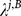, it is easy to find that for the original game two consecutive actions, when they are the same, determine the action of the next player and thus all of the remaining players. Therefore, one can study the game by simply enumerating all of the cases where cascade happens. However, there is no solid mathematical foundation here to claim that this blind action-counting approach is the best or the exact solution, although this approach is commonly used in analysis of the social learning games [2], [3], [6]–[8].

Another commonly used method is based on a Bayesian analysis of the game [9]–[14]. We will comment on these two solutions and demonstrate where the logical gaps exits in the two solutions in the next section.

The main contribution of this manuscript, in addition to showing the gaps in the two approximate solutions, is presenting an exact solution of our own. We will first present our calculation and then compare it against the two approximate solutions on the original game. Although as we will see later, our own solution is in principle different from the other two, we will see that there is almost no difference among the three solutions on the original social learning game. While we will also provide a reason of the same action outcomes from the three solutions, we propose a minor extension of the social learning game, to which all the solutions, if proper to the original game, should be applicable as well. We will, however, demonstrate that the three solutions lead to different average payoffs and that, on average, our exact solution has the highest payoff in the extended games. We believe this will be sufficient to illustrate that the two approximate solutions are not as good as the exact ones.

## Logical Gaps in the Blind Action-Counting and the Twisted Bayesian Approaches to the Social Learning Game

In the original definition of the social learning game, all learners know that the true status of the world follows a known priori distribution of all possibilities, which is usually taken as 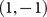: with a probability of 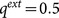 the status of the world is 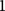, i.e.,
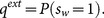
(2)However, after the world's status is initiated it stays at that status during the entire learning process. After the above-mentioned 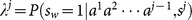 is known, the rest of decision making process is trivial, i.e. 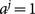 when 



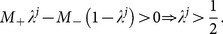
(3)and 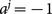 when 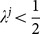. For the case of 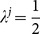 additional tie-breaking rules are required, for example 



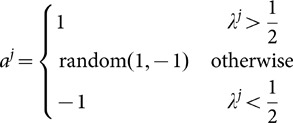
(4)Here 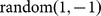 means to take one value from 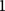 and 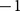 with equal probability. Other tie-breaking rules are also possible [4], [9]. This relation between 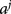 and 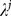 can also be denoted as 
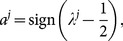
(5)which in the special case of 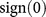 is assumed to be 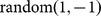 instead of its usual value 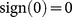. To use this relation between 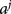 and 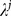 conveniently in later derivations, Eq. (4) can also be represented by a distribution function of 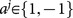 as 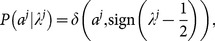
(6)where 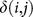 is the Kronecker 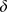 notation that it is 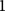 when 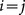 and 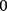 otherwise. One can check that Eq. (4), Eq. (5) and Eq. (6) are in fact the same even though the latter takes a form of probability distribution, 



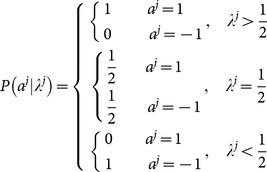
(7)The probability distribution form of Eq. (6) is very important in deriving our exact procedure, as we will see later.

Now that all of our terminologies and notations have been defined, let us start our discussions on solutions to the social learning game. We have mentioned that the only non-trivial part of the decision making process of the social learning game is the calculation of 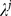. As we stated in the introduction, there are usually two approaches for this calculation. One is to simply count how many times action 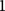
(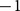) has been taken previously and denote it as 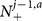
(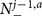) and compare the two values while taking into account her own private signal 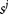, i.e. 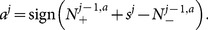
(8)From this formula, it seems that this decision making process does not really need 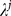. We will show later that in fact, it assumes a very special form of 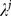
as in Eq. (22), and from which Eq. (8) can be derived. There we will see clearly what is missing in this argument. Here, we first want to present a count argument to note that there might be better decision making mechanisms than this blind action-counting approach.

Consider the case of a private signal sequence 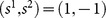 and the corresponding action sequence being 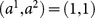, which is possible under the random tie-breaking rule. Up on observing this action sequence, according to the blind action-counting approach, the third learner will definitely choose the action 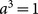 no matter what her private signal is. Assuming that her private signal is 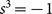, there is in fact a higher chance that the world is in state 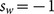 other than 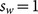. However, as argued above, the third learner will choose 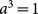 thus, future learners as well, leading to a wrong cascade.

Generally speaking when 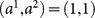 is observed, it more likely that the world is indeed in a state of 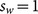, so it is not that wrong to choose action 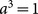. However, at least in principle, when the third learner gets 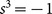, she should be more careful about simply discarding her own signal especially when she is fully aware of the random tie-breaking rule. Is there any possibility to take this into consideration? According to the blind action-counting approximate solution 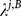, the answer is no. Will any other solutions be able to take care of this and do it better?

As we will see later, using the proper form of Bayesian analysis we can, in fact, do better: By figuring out all possible 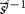 from observed 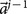, better decisions can be made.

The second commonly used approach [9]–[14] of calculation of this 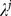 is more involved than counting 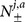. Let us rephrase the formula originally from [9] here in terms of our own notations. Assuming 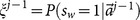 is known to the 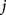
th learner for some reasons, which will be explained later, using Bayesian formula, we can have 



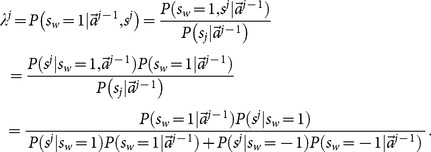
(9)This leads to 
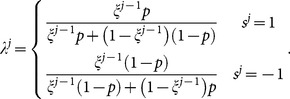
(10)This formula linking 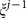 to 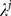, while it has a very confusing meaning as we will show latter, is mathematically sound. In order to form a closed formula system, it requires a formula linking 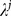 to 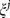, such that the next iteration will give 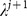, 
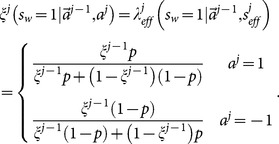
(11)In a sense, this assumes that upon observing 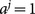, the 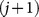
th learner will effectively think that 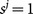 and similarly when observing 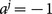. However, this step, exactly this step, is not necessary true.

To summarize, the above Bayesian analysis can be expressed as 



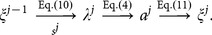
(12)Taking effectively that 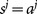 may not be that far off overall, is problematic and the exact source of the logical mistake of this approach. We call the above 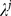 calculated by the twisted Bayesian approach especially from Eq. (11) the twisted-Bayesian approach and denote it as 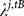. If we are going to follow this line of thinking we need a better formula from 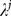 to 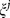.

There is another potentially misleading part in the above derivation of Eq. (9): While it is not mathematically wrong, letting 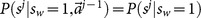 is logically not straight. In the left-hand side, we are thinking that knowing only the action history 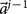 thus we need to figure out distribution of 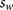 first according to this 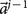, and then use this ‘figured out’ distribution of 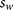 and limit ourselves in considering only the subset of 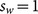, and then to calculate probability distribution of 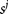 within the subset of 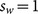; in right-hand side, we are thinking that when 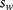 is known then the distribution of 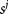 depends only on 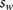 but not on 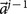. These two expressions are not at the same level of logic. One way of out of this insecure practice of mathematics is to completely avoid 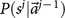 and consider instead 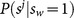 and 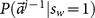, which are absolutely well-defined. We do so in the next section when constructing the exact formula of 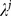.

The blind action-counting approach does not make use of the full information so that there are potentially rooms for better solutions and the twisted Bayesian analysis misses one important step in its mathematical formalism: There is no solid mathematical ground for Eq. (11), which links 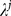 to 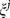.

In the rest of this manuscript, we will present a solution that makes use of the full information and also every step of it has a solid mathematical ground. The only catch is that it is quite mathematically involved and the idea originates from statistical physics, which might not be a common or familiar toolbox for researchers in social learning, game theory or even other fields of economics. In statistical physics, non-interacting systems are much easier to address and quite often, they provide a good starting point for building up formalism to tackle interacting systems. It is exactly this beauty of statistical physics that makes it possible to develop our own calculation of 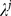.

## Exact Formula of 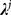


Using the Bayesian formula differently from Eq. (9), we can rearrange 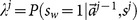 as 



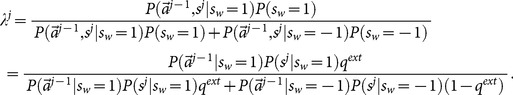
(13)Here, in the last step, we used the fact that the previous actions and the current private signal are two independent events. There is only one unknown term in Eq. (13)



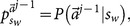
(14)and we denote it as 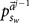, which is the probability of history of a specific previous 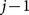 actions being 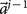, given world status 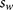.

Notice the above 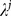 is subjective, i.e., it is in the 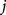
th learner's mind that how much she believes the status of the world is 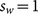 with given information 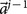 and 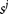. Therefore, 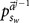 is also subjective. In the future, when calculating the rate of accuracy and the probability of cascading, we will need an objective probability 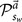. The way to find this 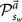 is to use 



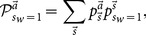
(15)where 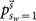 is totally objective and has nothing to do with learners' decision making and 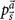 is the probabilities of all action outcomes 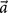 given signal sequence 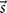 and depends on the learners' decision making. The way to calculate 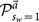 is to determine all action outcomes 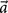 of a given 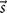 and then sum over all 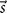 leading to the same 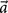 according to Eq. (15). To calculate 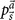, we need to generate all possible signal sequences 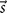 and go through the decision-making process, according to given approaches of calculating 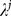, to determine action sequences 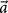 for each of the sequences 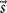. Notice that 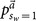 and 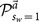 are potentially different.

### A simpler case where private signals are open to the public — finding 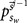


Calculating 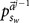 directly is not easy, however, calculating 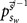 is straightforward, 



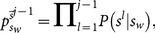
(16)where 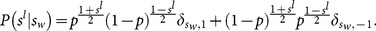
(17)Using the signal counting 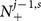 and 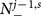, we arrive at 



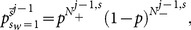
(18)

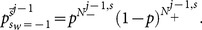
(19)In terms of these notations, if the private signals are available to the public, then upon receiving a signal 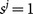 and providing 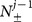, according to the Bayesian formula, 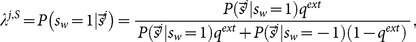
(20)the probability that the world's status is 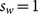 can be expressed as 



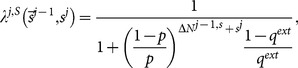
(21)where 
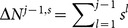
. When 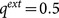, this probability is more than 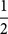 as long as 
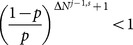
. It depends only on 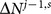. 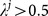 as long as 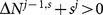. Similar procedure for this public-signal case has also been discussed in [15].

Next, we generalize the above calculation to a case where only the actions but not the private signals are known to learners.

### Blind action-counting 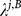


First, we want to spend a little bit of our time on revisiting of the blind action-counting approach. By revisiting it, we will clearly see what information is missing in the blind action-counting approach.

Even when only previous actions but not private signals are available to the public, let us assume for now that 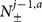
includes as much information as 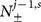, thus from Eq. (21) we have, 
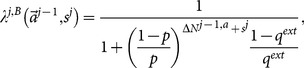
(22)where 
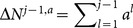
. Learners adopting this decision-making mechanism are regarding that action sequences provide as much information as signal sequences. This is obviously wrong. It can be shown that according to this formula, 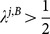 if 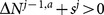 and therefore it lead exactly to Eq. (8).

In many previous studies of the social learning, calculations of probability of cascades and other quantities were based on this 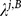 [2], [3], [6]–[8]. This is potentially sub optimal because action sequences are treated as reliable as signal sequences. Next, we are going to present one solution, in which signal sequences and their distributions are figured out first from action sequences and then decision is then made upon the inferred signal sequences.

### From 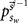 to 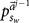 for exact 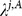


The idea is very simple. If we can turn the history of actions into a history of signals, we can make use of the above 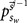 and then everything is done. That is to say, we want all possible signals 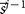 which lead to action 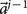. Making use of the law of total probability, we have 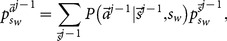
(23)where 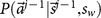 is related to the decision making process, and the decision making process does not directly involves 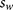 because 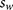 is not explicitly known to learners. Therefore, 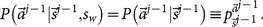
(24)We first notice that because we assume that learners make no mistakes, 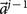 is fully determined by 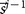 and the signal vector 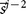 of previous learners has no direct effect on the current 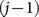
th learner's decision. Thus, 



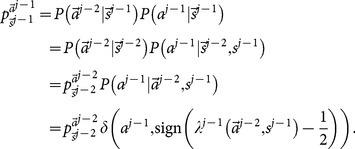
(25)In the second last step, we have used the fact that 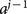 is not directly determined by 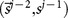, but is indirectly determined by 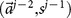, which in turn results from 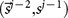.

Combining Eq. (25) and Eq. (23), we obtain 



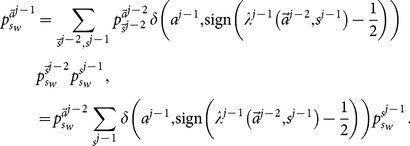
(26)Here we have used the fact that 
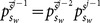
. Eq. (26) is the central formula of the present work.

Eq. (13) and Eq. (26) present an iterative procedure to find all 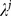, 



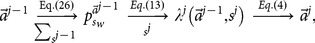
(27)where 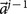, 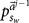 and the action outcome 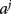 are public information, while the 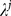 is private to the 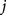
th learner since 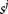 is hidden from the public. We call the 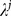 calculated from the procedure defined in Eq. (27) the exact solution 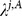 since it solve exactly the mathematical problem of finding 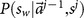. Here the Superscript 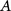 refers to the fact that it is calculated from histories of actions.

### Examples showing that 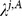 is potentially different from 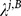 and 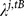


Next we illustrate one example of the results from the above iterative calculation 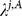 and the other two approximate solutions 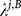 and 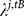. Assuming the private signals are 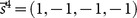, and when the second learner breaks the tie she ends up with 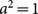, we get the 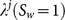
s and action outcomes listed in Table 1. Given this sequence of private signal, it is more likely the true state of the world is 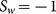. However, here we intentionally considered the case where the second learner chooses action 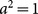 so that there is a chance of misleading the rest of the population.

**Table 1:** Possible action outcomes following different 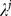
s. 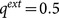, 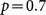.

	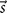	1	−1	−1	−1
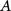	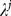	0.7	0.5	0.57	0.57
	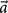	1	1	1	1
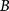	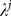	0.7	0.5	0.7	0.84
	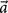	1	1	1	1
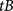	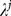	0.7	0.5	0.7	0.84
	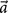	1	1	1	1

### A theorem on new criteria of cascades

Cascades have been a central topic in studies of social learning. With the idea behind 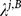, being to join the majority, the cascading problem becomes to identify the probability of two consecutive steps with the same actions occurring while the actions before the consecutive two steps are evenly distributed. This is in fact the working criteria of cascading used in many works including the well-known work of [3]. In this way, one gets the analytical results of the probability of a cascade before the end of the game 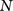, 
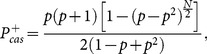
(28a)

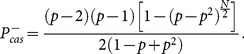
(28b)Instead of 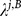, here we want to discuss the phenomenon of cascading based on our exact 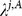. We will show that the event in which two consecutive learners take the same action is not a sufficient condition for cascading if decisions are made according to 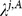.

**Theorem 1** *The social learning game starting from the* 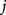 *th learner goes into a cascading state* 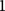 *(* 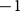 *) if and only if* 
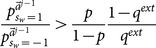
 *(* 
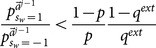
 *).*

**Proof**: *Cascading happens when* 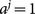 *(* 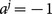 *) no matter what value the* 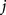 *th learner's private signal* 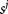 *is. First, we find the cascading condition for the* 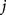 *th learner. In this proof without loss of generality, we consider only the case of* 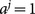 *. From Eq. (13), we know* 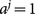 *is equivalent to* 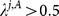 *. Considering the case* 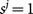 *and* 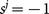 *, that condition becomes respectively*



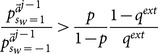
(29)

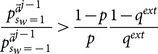
(30)
*Eq. (30) is naturally satisfied if Eq. (29) is. So the condition holds if and only if Eq. (29) is true. Second, we want to show that whenever this occurs for the* 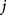 *th learner, the same holds for the* 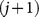 *learner. From Eq. (26), given that* 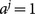 *, then*



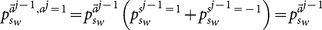
(31)
*Therefore, Eq. (29) is again satisfied for* 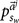 *.*

From this theorem, we know that cascading happens whenever the action taken by a learner does not depend on its private signal. Furthermore, this theorem shows that whenever that happens, later learners can not change this trend of cascading. We call such a sequence of actions 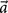 as cascading 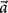. This is different from the prior criteria of cascading [3] being that two consecutive learners take the same action while the actions before the two were evenly divided between the two actions. In fact, if 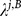 is plugged into Eq. (29), rather than the exact 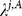, we arrive at the prior criteria. However, when 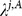 is used, such criteria is no longer sufficient because even after such “criteria” are met it is possible for the next learner to jump out of the old “cascade”. In fact, this is intuitively why the rate of accuracy based on 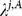 is higher than that of 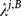.

### Comparison between the exact and the two approximate solutions

With the exact decision making procedure 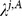 equipped to every learner, let us now discuss action outcomes of this social learning game. Given values of 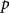 and 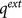, we define as the rate of accuracy as 
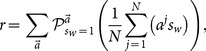
(32)where the product 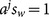 when action 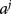 is the same as 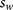. This 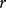 describes average payoff of all learners in a game. 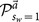 is the objective probability of the action series 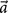 as discussed in Eq. (15). Next we will compare this average payoff of the three decision making procedures according to, respectively 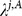, 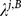 and 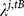.

We are also interested in the whole probability of cascading towards respectively 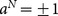, i.e. those action sequence 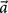 satisfying that either 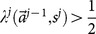 or 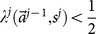 no matter 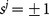 for a given 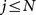, 
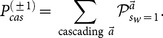
(33)Cascades mean that a learner's action does not depend on her private signal.

Here, all numerical results will be presented only for the case of 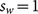 since the cases of 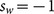 and 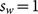 are exactly symmetric. For reasons that will be clear later, all reported results in the following are from games with 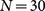, if not explicitly stated.

**Figure 1:**
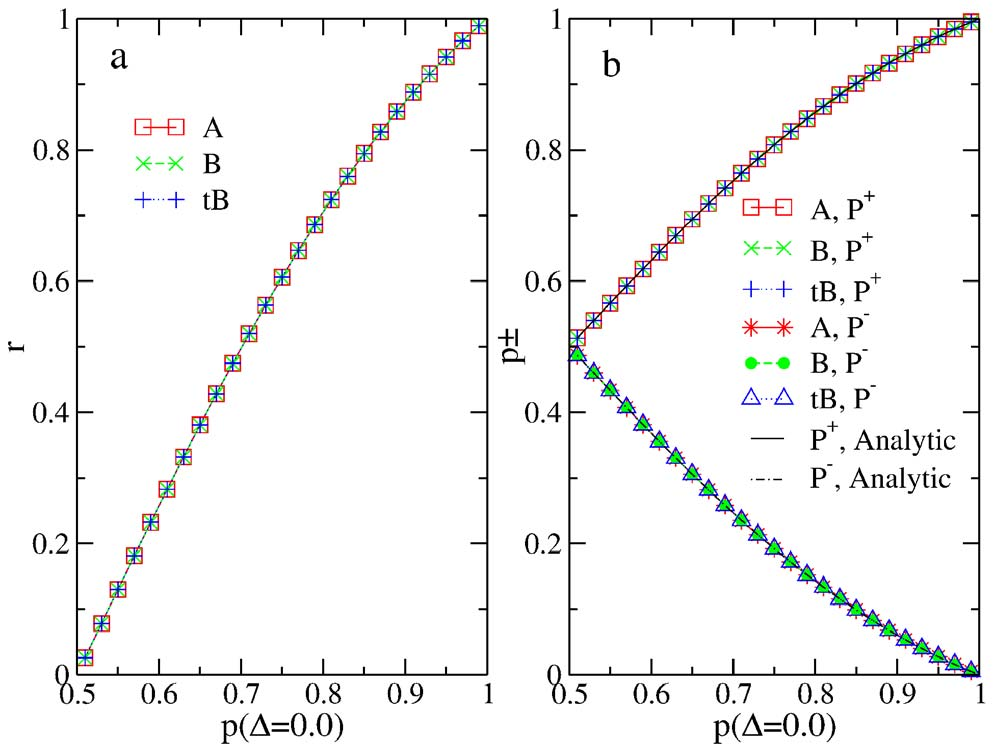
Rate of accuracy (a) and probability of cascading before 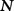 (b) of the original RD models studied using the exact 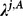, compared with the blind 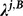 and the twisted Bayesian 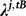. Results from the three calculations are exactly on top of each other. In (b), analytical results from Ref. [3] are also plotted. 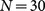 in this and other figurers, unless noted otherwise.

From Fig. 1, we can see that there is no difference on respectively the rates of accuracy (a) or the probabilities of cascading(a) at all from all three solutions. In Fig. 1(b), analytical results from [3] are plotted and are shown to exactly agree with our numerical results.

This finding agrees with our example in the previous subsection: Although the numerical values of 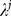
s from the three solutions can be different, the action outcomes are always the same because the three solutions agree with each other on whether 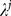 is larger or smaller than 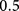. Next, we want to further illustrate that in more general situations these different numerical values may lead to different actions.

## Minor Modifications of the Game and Difference between the Exact and the Approximate Solutions

We have seen that although both 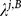 and 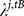 are only approximate solutions while 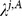 provides the exact solution of the mathematical problem of finding 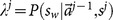 and these solutions have different numerical values, when applied to real games, there is no difference among the three solutions. Why? The different numerical values agree on being larger or smaller than 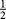, although their difference from 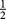 are different. Due to this observation, here we propose some slight extensions of the game. The extension is so marginal that in principle, if all of them are proper solutions of the original game, the three solutions should also be proper solutions to the extended game. We will, however, show that on the extended game there are visible differences among results of the three solutions.

We introduce a parameter 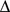 to represent reservation of a learner to take an action, i.e. given 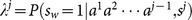, a learner 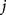 can make a decision according to 
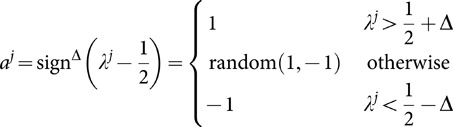
(34)We take 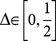. Such a reservation can be related with a service charge of taking actions in real game playing. In that case, allowing the learners to take no actions in their turn when 
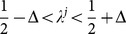
, i.e. replacing 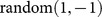 with simply 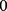, makes even better sense. However, we will not discuss this additional modification or the motivation of introducing this 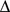 here. What we want to argue is that nothing forbids the applicability of each of the three above 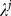
s to this extended model if they are indeed applicable to the original one, where simply 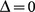 and is the only difference between the extended and the original games. From where we stand, we simply want the different numerical values of 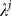 from the three solutions to make a difference on action outcomes.

Next, we want to compare results of the three solutions on the extended model with 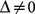.

### 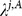 is different from 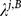 and 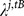 when 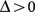


Above we have shown that although the resulted formulae and the numerical values are different among 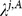, 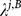 and 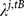, the resulted actions are not different at all when 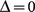. Now let us do the same comparison when 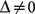: Table 2 shows the numerical values of 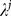
s and action outcomes in the case of 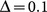. The third learner chooses to act randomly according to 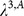 and chooses action 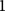 according to 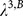 and 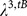. This randomness in the third learners' actions leads to a chance of correcting the unintentional and undesired actions of the second learner (being 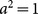), which will lead to a wrong cascade when solutions 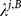 or 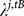 is used. This chance of correction makes it possible to generate higher payoffs for the rest of the population.

**Table 2:** Possible action outcomes following different 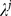
s. 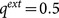, 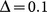, 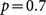.

	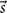	1	−1	−1	−1
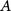	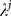	0.7	0.5	0.57	0.36
	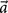	1	1	−1	−1
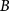	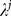	0.7	0.5	0.7	0.84
	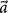	1	1	1	1
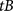	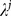	0.7	0.5	0.7	0.84
	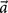	1	1	1	1

**Figure 2:**
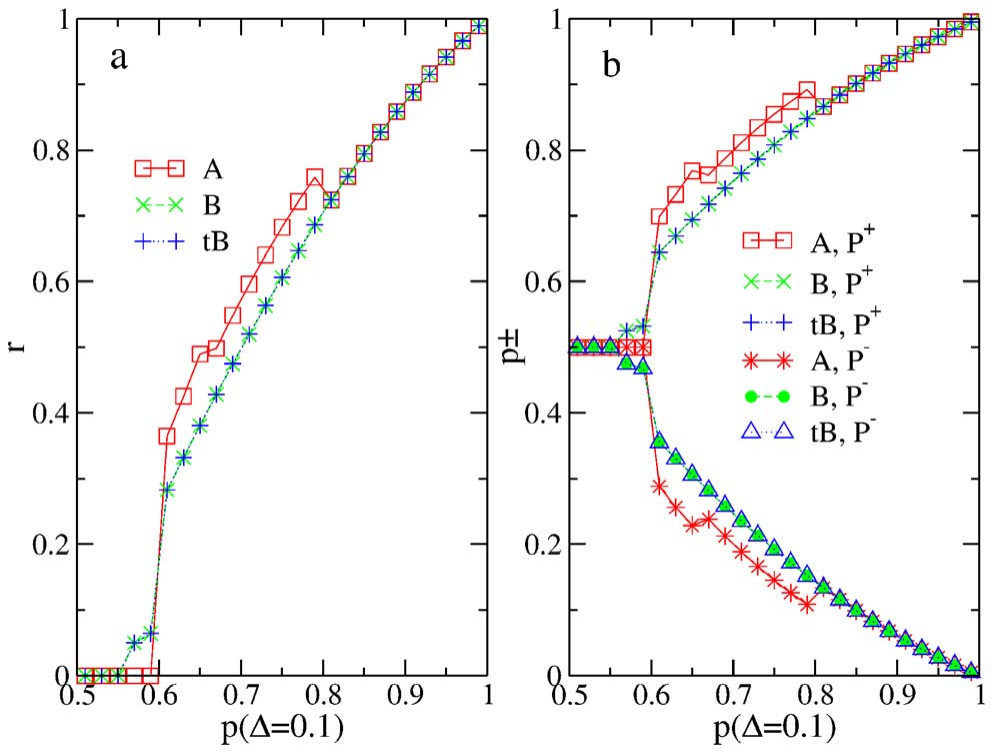
Rate of accuracy (a) and probability of cascading before 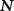 (b) of the extended model with 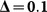 solved by the exact 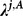, the blind 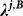 and the twisted Bayesian 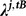. Results from 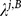 and 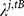 are still on top of each other, while in some cases, 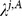 leads to better accuracy.

We also see manifestation of this difference in simulation results. Where 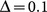, we see that rate of accuracy and the probability of cascading calculated from 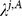, shown in Fig. 2, are different from and in fact higher than those calculated from 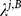 and 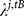. Therefore, action sequences from the strategic state 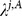 must be different from those from 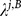 or 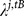.

### 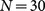 is large enough

**Figure 3:**
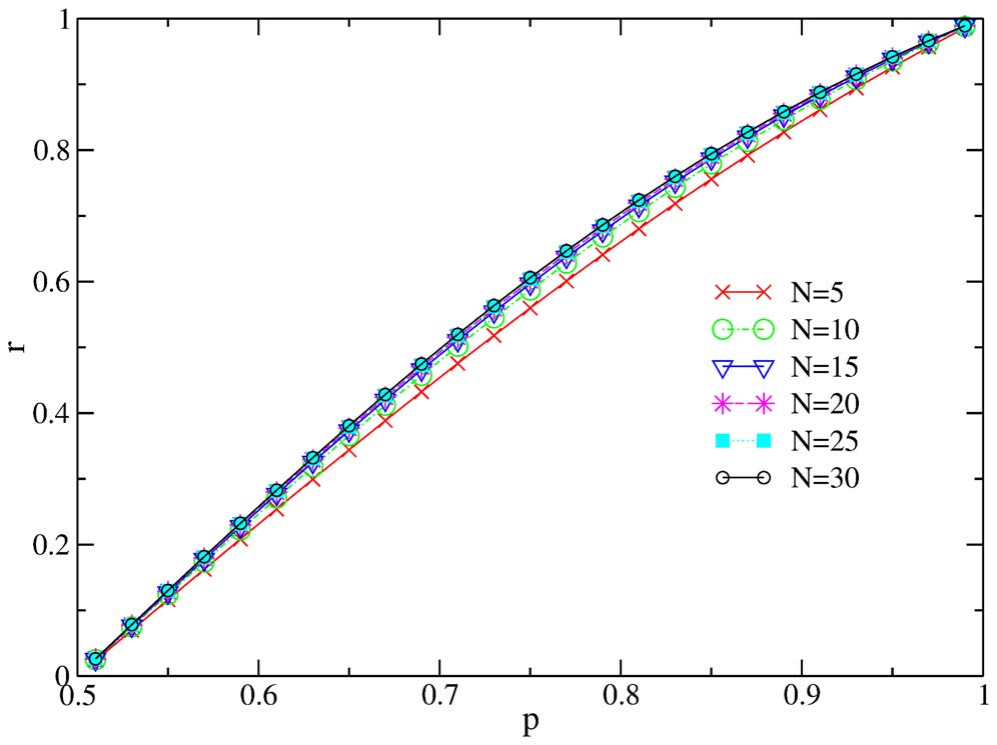
Rate of accuracy found from the exact solution for various values of 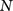. We see that as long as 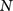 is large enough, there is no visible difference on the rate of accuracy for various 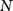. 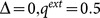.

We have argued earlier that due to the fact that because a cascade quite often happens after only a few learners taking their actions, it is not necessary to run this simulation of social learning games for a very large population size 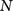. To test this further, we plot the rate of accuracy found from the exact solution for various values of 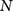 in Fig. 3. This plot shows that the difference between 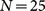 and 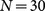 is very small. This confirms that it was reasonable to set 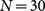 for all previous game results.

## Conclusions

In this work, an exact solution to the original social learning game is proposed and logical gaps in the two approximate and commonly used solutions are discussed. To demonstrate that our own solution is not only different in principle but also lead to different outcomes in actions and payoffs when compared with the other two solutions, we modified the game to incorporate parameter 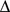, which stands for level of reservation for risk taking. Our calculations and simulations indeed show that higher payoffs can be achieved in this case when using the exact solution.

With this exact solution, other essential questions in the social learning game, such as conditions of correct information cascades and mechanisms to improve probability of correct information cascades, should all be re-analyzed. Recently, there are extension of the social learning game to consider effect of complex networks [16] and changing environments [9]. Discussions of all such extended models should also in principle be analyzed using the exact solution.

We have also confirmed via the example calculation in Table 2 and the above numerical simulations that action outcomes and thus the received payoffs from 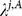 are different from outcomes from 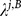 or 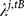 in the extended model.

It is very costly, however, for a learner to really implement the exact solution, in that a large amount of tricky mathematical calculation is required to adopt 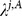. How close are results from our human decision making processes, such as a dynamical process of public opinion, to the exact solution? This is another interesting question. A trivial solution to this is that once learners understand that a more mathematically involved formula is needed to play the social learning game, they can always use a computer to help them in their decision making. However, in the real world, should we expect that action outcomes to be close to those predicted by our exact solution or the other two? This highly non-trivial question remains and should be a question for further investigation.

## References

[1] Bikhchandani S, Hirshleifer D, Welch I (2008) information cascades. In: Durlauf SN, Blume LEeditors, The New Palgrave Dictionary of Economics, Basingstoke: Palgrave Macmillan.

[2] Bikhchandani S, Hirshleifer D, Welch I (1998) Learning from the behavior of others: Conformity, fads, and informational cascades. Journal of Economic Perspectives 12:151–170.

[3] Bikhchandani S, Hirshleifer D, Welch I (1992) A theory of fads, fashion, custom, and cultural change as informational cascades. Journal of Political Economy 100: pp. 992–1026.

[4] Banerjee AV (1992) A simple model of herd behavior. Quartely Journal of Economics 107:797–817.

[5] Szolnoki A, Wang Z, Perc M (2012) Wisdom of groups promotes cooperation in evolutionary social dilemmas. Scientific Reports 2:576. 22893854 10.1038/srep00576 PMC3418638

[6] Callander S, Hoerner J (2009) The wisdom of the minority. Journal of Economic Theory 144:1421–1439.

[7] Guarino A, Harmgart H, Huck S (2011) Aggregate information cascades. Games and Economic Behavior 73:167–185.

[8] Neill DB (2005) Cascade effects in heterogeneous populations. Rationality and Society 17:191–241.

[9] Moscarini G, Ottaviani M, Smith L (1998) Social learning in a changing world. Economic Theory 11:657–665.

[10] Smith L, Sorensen P (2000) Pathological outcomes of observational learning. ECONOMETRICA 68:371–398.

[11] Bose S, Orosel G, Ottaviani M, Vesterfund L (2006) Dynamic monopoly pricing and herding. RAND Journal of Economics 37:910–928.

[12] Bose S, Orosel G, Ottaviani M, Vesterlund L (2008) Monopoly pricing in the binary herding model. Economic Theory 37:203–241.

[13] Gill D, Sgroi D (2008) Sequential decisions with tests. Games and Economic Behavior 63:663–678.

[14] Liu T, Schiraldi P (2012) New product launch: herd seeking or herd preventing? Economic Theory 51:627–648.

[15] Easley D, Kleinberg J (2010) Networks, Crowds, and Markets: Reasoning About a Highly Connected World. Cambridge University Press.

[16] Acemoglu D, Dahleh MA, Lobel I, Ozdaglar A (2011) Bayesian learning in social networks. Review of Economic Studies 78:1201–1236.

